# Acute-onset endophthalmitis following pars plana vitrectomy for symptomatic vitreous opacities: a case series

**DOI:** 10.1186/s40942-026-00877-4

**Published:** 2026-07-03

**Authors:** Michael Y. Zhao, Jin Kyun Oh, Landon J. Rohowetz, Marc H. Levy, Jody Abrams, Curtis E. Margo, Steven Cohen, Jorge Fortun, William E. Smiddy, Stephen G. Schwartz, Harry W. Flynn

**Affiliations:** 1 Department of Ophthalmology, Bascom Palmer Eye Institute, University of Miami Health System, 900 NW 17th St, Miami, FL 33136 USA; 2 Sarasota Retina Institute, Orlando, FL USA; 3 Department of Ophthalmology, Morsani College of Medicine, Tampa, FL USA; 4 Department of Pathology and Cell Biology, Morsani College of Medicine, Tampa, FL USA; 5 Retina Vitreous Associates of Florida, Clearwater, FL USA

**Keywords:** Pars plana vitrectomy, Vitreous opacities no-light-perception, Acute-onset endophthalmitis, Patient outcomes

## Abstract

**Background:**

Pars plana vitrectomy (PPV) for symptomatic vitreous opacities, commonly referred to as floaterectomy, is an increasingly performed elective procedure that can meaningfully improve quality of life. However, like all intraocular surgery, it carries the risk of endophthalmitis. To our knowledge, only 2 cases of this complication following floaterectomy have previously been reported. The purpose of this study is to report the largest series of acute-onset bacterial endophthalmitis following floaterectomy, characterizing clinical presentation, causative organisms, antibiotic susceptibilities, histopathology, and visual outcomes.

**Methods:**

This was a retrospective, interventional case series conducted at a tertiary referral center. A query of all patients who underwent PPV for vitreous opacities from January 2010 through December 2025 was performed. Patients were included if they developed acute-onset endophthalmitis (within one month of PPV).

**Results:**

Five eyes of 5 patients were included. Two of 394 patients (0.5%) who underwent floaterectomy at our institution developed acute-onset endophthalmitis; 3 additional cases were referred from outside retina specialists. Mean presenting BCVA was 2.1 logMAR (~ 20/2500). Four of 5 vitreous cultures (80%) were positive: 2 gram-positive (*Staphylococcus caprae*, *Staphylococcus lugdunensis*) and 2 gram-negative (*Pseudomonas aeruginosa*, *Serratia marcescens*). The institutional endophthalmitis rate of 0.5% was not statistically different from published post-PPV rates of 0.16–0.28% (*P* = 0.13). Visual outcomes were polarized: 3 of 5 eyes (60%) achieved BCVA ≥ 20/80, while 2 of 5 (40%) progressed to no light perception and underwent enucleation, both involving gram-negative organisms. Histopathology in one enucleated eye demonstrated extensive intraocular necrosis and suppurative inflammation.

**Conclusions:**

Acute-onset endophthalmitis following floaterectomy is rare, occurring at a rate statistically similar to PPV for other indications. However, given that floaterectomy is elective surgery for a largely benign, non-vision-threatening condition, the harm-to-benefit ratio is inherently less favorable than PPV performed for sight-threatening pathology. Gram-negative infections were associated with catastrophic and irreversible vision loss, including enucleation. These findings underscore the importance of thorough, candid preoperative counseling regarding the risk of severe infectious complications before proceeding with floaterectomy.

## Background

Symptomatic vitreous opacities (i.e., floaters) are relatively benign but may cause variable visual symptoms that are not captured by Snellen visual acuity testing. While these are usually tolerable symptoms, their impact may sufficiently impact patients’ visual function that removal via pars plana vitrectomy (PPV), often referred to as floaterectomy, is considered to improve quality of life [1]. Subjectively, it is our impression that such patients are almost always very satisfied postoperatively [2, 3]. Boneva et al. reported limited refractive vitrectomy may lead to improved level of visual function and patient happiness, but mentioned observation without treatment is also a viable management strategy [4]. Small incision surgery and improved instrumentation have reduced complications [5]. Therefore, surgery for vitreous opacities has become more frequently performed [5, 6]. 

However, any intraocular surgical procedure carries the risk of endophthalmitis. To our knowledge, there have only been 2 reported cases of acute-onset endophthalmitis following floaterectomy, one of which was from our institution [7, 8]. While postoperative endophthalmitis is a rare (~ 0.16–0.28%) complication of PPV, there is a limited information on treatment outcomes for acute-onset endophthalmitis following PPV for vitreous opacities [9, 10]. This case series describes the clinical presentation, antibiotic susceptibility, treatment outcomes, and histopathology in five patients.

## Methods

Institutional Review Board (IRB)/Ethics Committee approval was obtained. This study adhered to the tenets of the Declaration of Helsinki. Written informed consent was obtained from all patients for publication of the clinical details. A review was performed of all patients who underwent pars plana vitrectomy (CPT code 67036) for vitreous opacities (ICD-10 code H43.39) at the University of Miami, Bascom Palmer Eye Institute from January 2010 through December 2025. Patients were included if they were diagnosed with acute-onset endophthalmitis (ICD-10 code H44.0) within 1 month of PPV. In addition, the medical records of referred patients who developed endophthalmitis after PPV for symptomatic vitreous opacities were included in this study. Fisher’s exact test was used to compare the institutional endophthalmitis rate with published post-PPV endophthalmitis rates.

## Results

**Fig. 1:**
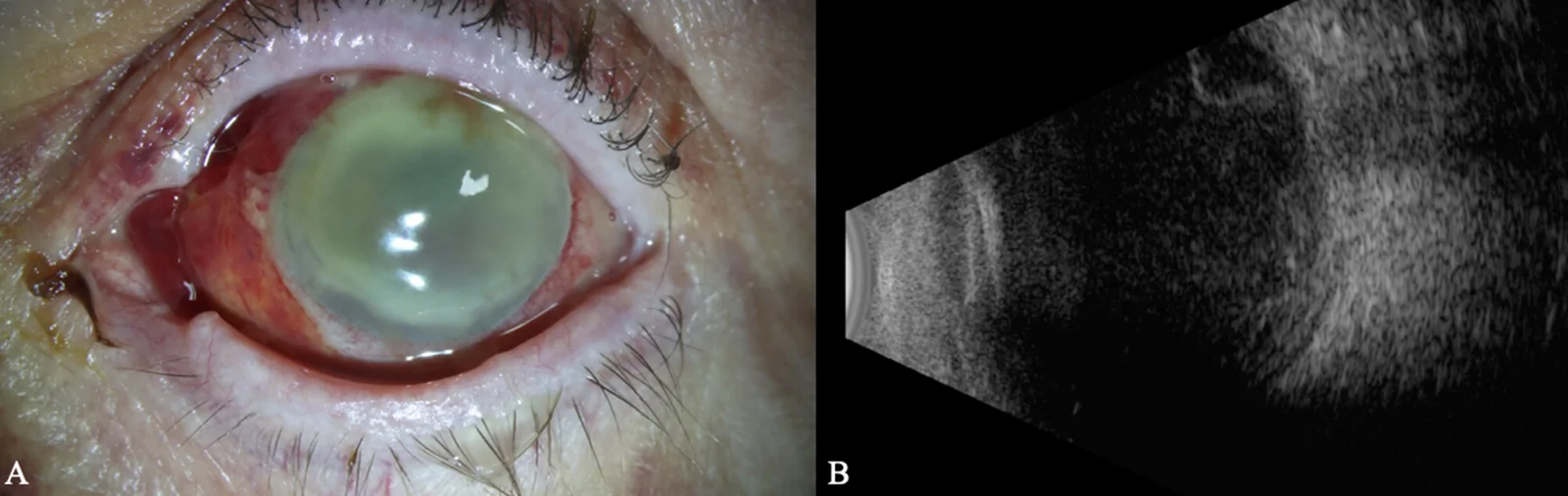
Patient 1 left eye. (**A**) Slit lamp photography showing diffuse injection, corneal ring infiltrate, and hypopyon. (**B**) B-scan ultrasound showing vitreous opacities and early membrane formation. Despite treatment, best corrected visual acuity at last follow-up was no-light-perception and the eye underwent enucleation

Five patients met inclusion criteria. Two of 394 (0.5%) patients who underwent PPV for symptomatic vitreous opacities at Bascom Palmer Eye Institute were diagnosed with acute-onset endophthalmitis. Three patients were referred to Bascom Palmer Eye Institute from outside providers. The mean age was 59 years (median 67 years), and 2 were men. To the best of our knowledge, none of the patients had a history of prior intraocular procedures, including cataract surgery or intravitreal injections; ocular comorbidities such as mild cataract and mild diabetic retinopathy were present in some patients but were under observation without active intervention. In all cases, they presented clinically with painful loss of vision, conjunctival injection, hypopyon, dense anterior chamber cell and/or fibrin with or without posterior segment opacities, such as vitreous cells, vitritis, and/or membranes (Fig. 1). Clinical data of presentation and initial treatments are summarized in Table 1. Of note, patient 3 was previously reported; her clinical data were included in this study [7]. Clinical data of additional treatments and visual outcomes are described in Table 1. Best corrected visual acuities (BCVAs) were polarized at last follow-up: *≥*20/80 in 3 patients, and NLP leading to enucleation in 2 eyes.

**Table 1:** Clinical presentations and initial treatments of patients with acute-onset endophthalmitis following PPV for symptomatic vitreous opacities

Patient	Age/Sex/Eye	BCVA @ Presentation	Initial Treatments	Organism Isolated	Clinical Course/Comorbidities	Additional Treatments	BCVA @ Last Follow-up
1	67/F/OS	LP @POD#2	IV van, cef @POD#2	*Pseudomonas aeruginosa*	Developed RD @POD#3 OS became NLP and painful	PPV/SB/SOI @POD#3 Enucleation @POW#3	Enucleated
2	71/F/OS	LP @POD#1	IV van, cef, dex @POD#1 PPV and repeat IV ami, cef, dex @POD#3	*Serratia marcescens*	OS became NLP and painful	Enucleation @POW#2	Enucleated
3	24/F/OD	5/200 @POD#1	Topical ciprofloxacin, prednisolone acetate, cyclopentolate @POD#1 IV van, cef, dex @ POD#2 and oral moxifloxacin 400 mg BID and topical besifloxacin	*Staphylococcus caprae*	Developed nuclear sclerosis	N/A	20/80
4	70/M/OD	LP @POD#3	PPV/1000cs SO with IV van, ami, dex @POD#3	*Staphylococcus lugdunensis*	Allergic to amoxicillin Developed recurrent CME @POM#6	PPV/SOR/AC washout @POW#6	20/30
5	63/M/OS	20/70 @POD#2 (baseline 20/20)	IV van, cef @POD#2	No organism isolated	Mild diabetic retinopathy and macular edema	N/A	20/20

Abbreviations: BCVA, best corrected visual acuity; Cef, ceftazidime; cs, centistokes Dex, dexamethasone; HM, hand motion; IV, intravitreal; LP, light perception; OD, right eye; OS, left eye; POD, post-operative day; POM, post-operative month; POW, postoperative week; PPV, pars plana vitrectomy; RD, retinal detachment; RLF, retained lens fragment; SB, scleral buckle; SO, silicone oil tamponade; SOI, silicone oil injection, SOX, silicone oil exchange Van, vancomycin

At the initial examination, mean BCVA was 2.1 logMAR (~ 20/2500; SD 0.9), and the median was 2.7 logMAR (light-perception; IQR 1.2–2.7). Time to presentation was between 12 h and 5 days following PPV; patient 2 presented within 12 h. At last follow-up for the 3 sighted eyes, the mean BCVA was 0.26 logMAR (~ 20/36; SD 0.3), and the median was 0.18 logMAR (~ 20/30; IQR non-applicable).

Four bacteria were isolated from 5 vitreous samples. Antibiotic susceptibilities and minimum inhibitory concentrations (MICs) in mcg/ml are described in Table 2. The two Gram-negative organisms (*Pseudomonas aeruginosa* and *Serratia marcescens*) were both sensitive to ceftazidime and amikacin. Eyes that isolated gram-negative organisms ultimately had no-light-perception. The gram-positive organisms (*Staphylococcus caprae* and *Staphylococcus lugdunensis*) were sensitive to vancomycin. At last follow-up, the BCVAs were 20/80 and 20/30 for the patients who isolated *Staphylococcus caprae* and *Staphylococcus lugdunensis*, respectively.

**Table 2:** Antibiotic susceptibilities and minimum inhibitory concentrations (mcg/ml) of patients with acute-onset endophthalmitis following floaterectomy

Patient #	Vancomycin	Gentamicin	Ceftazidime	Oxacillin	Moxifloxacin	Clindamycin	Amikacin
1	N/A	N/A	S (2)	N/A	S (≤ 0.25)	N/A	S (2)
2	**N/A**	**S (≤** **1)**	**S (≤** **1)**	**N/A**	**S (≤ 0.12)**	**N/A**	**S (≤** **2)**
3	S	N/A	N/A	N/A	S	N/A	N/A
4	S (1)	I (8)	N/A	R (≥ 4)	S (≤ 0.25)	S (0.25)	N/A

Abbreviations: I, Intermediate; R, Resistant; S, Sensitive. Minimum inhibitory concentration, if available, is in parentheses in mcg/ml

## Discussion

The rate of endophthalmitis following PPV for floaters at our institution was 2/394 (0.5%) which is not statistically different from reported rates of endophthalmitis following PPV for all diagnoses (*P* = 0.13) [9, 10]. However, the reported visual and clinical outcomes as well as microbiology of endophthalmitis following retina surgery (e.g., PPV) were reported to be worse than endophthalmitis caused by other intraocular procedures [11, 12]. In the current study, exam findings of endophthalmitis following PPV for vitreous opacities (e.g., hypopyon, painful loss of vision) were similar to those cases of endophthalmitis from PPV in general [11]. 

Reported visual outcomes for endophthalmitis after vitrectomy vary widely in the literature. In a prospective study, Park et al. reported 29.6% of eyes with post-PPV endophthalmitis were eviscerated or had NLP vision while 14.8% regained BCVA > 20/40 [11]. In a retrospective study, Bhende et al. reported 63.2% of eyes with post-PPV endophthalmitis attained a BCVA of < 5/200 at last follow-up while 13.2% regained BCVA > 20/60. Bhende et al. also reported eyes that endophthalmitis caused by gram-negative organisms had poorer outcomes compared to those caused by gram-positive organisms. However, the results of the current study, while consistent with post-PPV endophthalmitis, in the context of floaterectomy, is particularly sobering since preoperative visual acuity is generally so good. Floaterectomy is an elective procedure; floaters are a largely benign condition. Thus, the harm-to-benefit ratio is inherently less favorable, and the potential for irreversible vision loss must be considered carefully in the preoperative setting. The favorable outcome in patient 5, despite negative vitreous culture, may reflect an early treatment effect, a low-inoculum infection, or involvement of a less virulent organism; however, definitive conclusions cannot be drawn given the absence of microbiologic confirmation.”

**Fig. 2:**
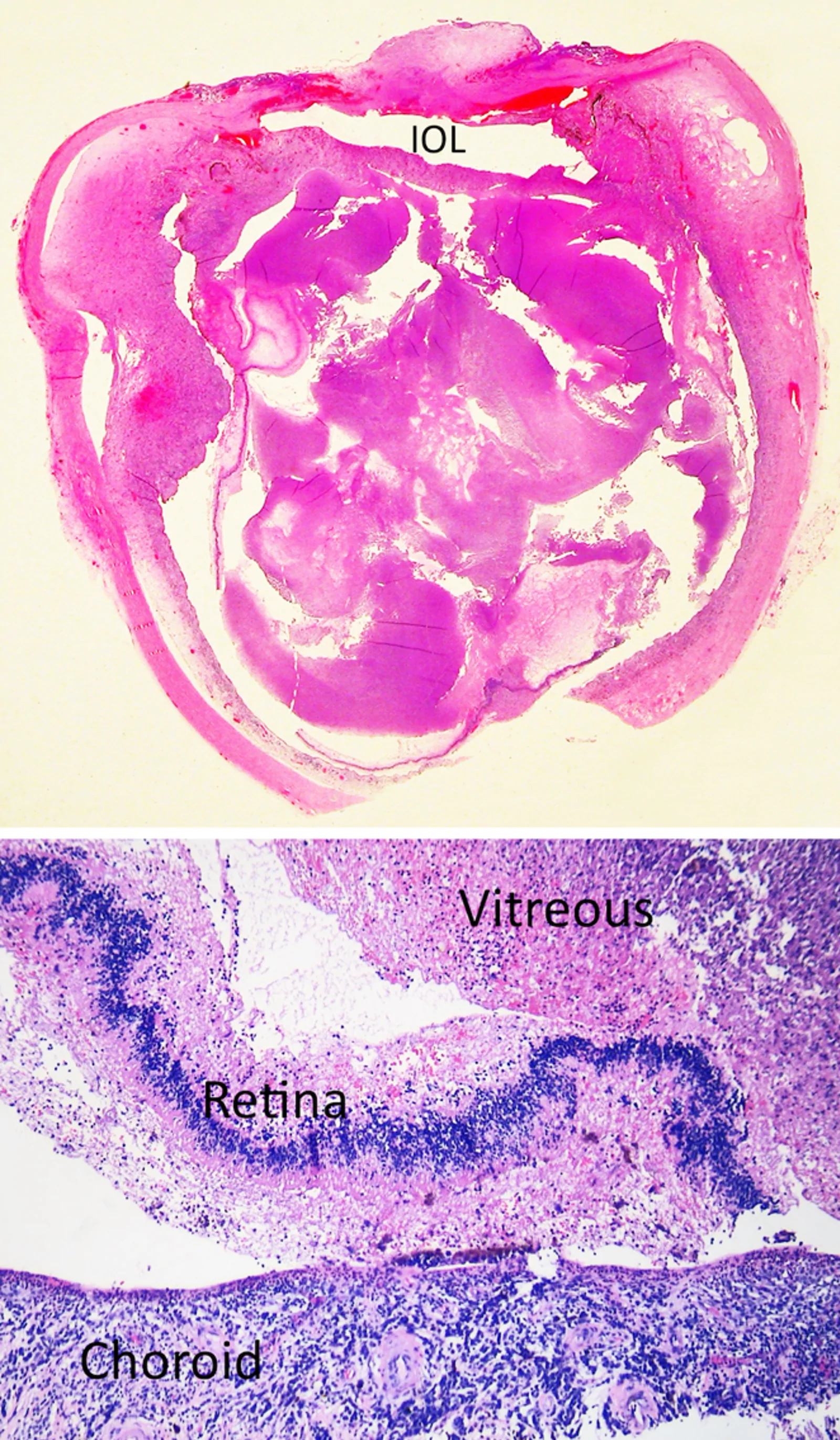
Left eye from a 71-year-old woman (patient 2). She presented 1 day after pars plana vitrectomy for symptomatic vitreous opacities in the left eye with visual acuity of light perception. (**A**) Whole mount of enucleated left eye demonstrating suppurative necrosis of the peripheral cornea with intraocular exudate spreading into limbal conjunctiva, intense intraocular inflammation and purulence, and suppurative encased intraocular lens. (**B**) Vertical cross section demonstrating purulent inflammation of vitreous, extensive retinal necrosis, and intense inflammation of the choroid

Multiple studies have reported high percentages (~ 14–20%) of gram-negative bacteria, especially *Pseudomonas*, as the isolated organism in endophthalmitis following PPV for any indication [11–13]. Gram-negative organisms are generally associated with a more aggressive clinical course; these bacteria produce endotoxins (lipopolysaccharides) and other cytotoxic factors that may trigger intense inflammatory responses, leading to severe damage to intraocular structures, as evidenced by the extensive necrosis in Fig. 2 [14]. While all gram-negative endophthalmitis cases are often associated with poor outcomes, those caused by *Pseudomonas* species are particularly severe. For instance, Sridhar et al. in 2015 reported 92% of eyes with *Pseudomonas* endophthalmitis (mostly after cataract surgery or post-penetrating keratoplasty) had a BCVA of light perception or worse, and 42% underwent enucleation [15]. Furthermore, *Serratia*, while less common than other gram-negative organisms, is recognized as a cause of post-procedural endophthalmitis and is associated with poor visual outcomes [16]. Another retrospective study by Sridhar et al. reported 60% of patients with endophthalmitis after cataract surgery caused by *Serratia marcescens* had a post-treatment BCVA of NLP at last follow-up [16]. In contrast, gram-positive bacteria more commonly cause a more indolent infection with better visual outcomes, unless caused by enterococci or streptococci species [17]. 

## Limitations

Findings of the current study are subject to inherent limitations applicable to all retrospective studies. The small sample size (*n* = 5) precludes conclusions about whether outcomes specifically after floaterectomy-associated endophthalmitis differ from post-PPV endophthalmitis for other indications. Furthermore, this retrospective case series is subject to biases of non-standardized management, documentation, and follow-up regimen. It is unknown how many PPVs were performed for symptomatic vitreous opacities and what the exact surgical techniques were performed by outside vitreoretinal surgeons, which may have represented selection bias.

## Conclusion

The rate of endophthalmitis following PPV for symptomatic vitreous opacities was not statistically significantly different from that of PPV for other indications. However, since floaterectomy is an elective surgery, the harm-to-benefit ratio is fundamentally less favorable. The potential for irreversible vision loss, including enucleation, must be weighed even more cautiously against the relief of a symptom that, while impactful, does not itself threaten vision. Nevertheless, there are patients who can attain visual benefit from removal of vitreous opacities. This underscores the importance of thorough, candid preoperative counseling that explicitly addresses the possibility of severe infectious complications before proceeding with floaterectomy.

## Data Availability

The datasets used and/or analyzed during the current study are available from the corresponding author on reasonable request.

## Declarations

### Publication originality statement

We confirm this publication is original.

## Abbreviations

BCVA: Best corrected visual acuity
Cef: Ceftazidime
cs: Centistokes Dex, dexamethasone
HM: Hand motion
IV: Intravitreal
LP: Light perception
OD: Right eye
OS: Left eye
POD: Post-operative day
POM: Post-operative month
POW: Postoperative week
PPV: Pars plana vitrectomy
RD: Retinal detachment
RLF: Retained lens fragment
SB: Scleral buckle
SO: Silicone oil tamponade
SOI: Silicone oil injection, SOX, silicone oil exchange Van, vancomycin

## References

[1] Duke RCT, Fischer NA, Crosson JN, Albert MA, Feist RM, Mason JO. Long-term outcomes on quality of life following sutureless vitrectomy for symptomatic vitreous floaters. J Vitreoretin Dis. 2025. 10.1177/24741264251388103. PubMed PMID: 41208950; PubMed Central PMCID: PMC12588966. PMC12588966 41208950

[2] Schwartz SG, Flynn HW, Fisher YL. Floater scotoma demonstrated on spectral-domain optical coherence tomography and caused by vitreous opacification. Ophthalmic Surg Lasers Imaging Retina. 2013;44(4):415–8. 10.3928/23258160-20130715-14 . PubMed PMID: 23883538. 23883538

[3] Gill N, Singh A. Management of Visually Significant Vitreous Opacities. Mo Med. 2024;121(5):391–4. PubMed PMID: 39421475; PubMed Central PMCID: PMC11482840. PMC11482840 39421475

[4] Boneva SK, Nguyen JH, Mamou J, Yee KM, Hoerig C, Silverman RH, et al. Clinical Management of Vision Degrading Myodesopsia from Vitreous Floaters: Observation vs. Limited Refractive Vitrectomy. Ophthalmol Retina. 2025;9(12):1149–58. 10.1016/j.oret.2025.05.014 . PubMed PMID: 40381842; PubMed Central PMCID: PMC12354178. PMC12354178 40381842

[5] Mason JO, Neimkin MG, Mason JO, Friedman DA, Feist RM, Thomley ML, et al. Safety, efficacy, and quality of life following sutureless vitrectomy for symptomatic vitreous floaters. Retina Phila Pa. 2014;34(6):1055–61. 10.1097/IAE.0000000000000063. PubMed PMID: 24384616. 24384616

[6] Schiff WM, Chang S, Mandava N, Barile GR. Pars plana vitrectomy for persistent, visually significant vitreous opacities. Retina Phila Pa. 2000;20(6):591–6. 10.1097/00006982-200011000. -00001 PubMed PMID: 11131410. 11131410

[7] Henry CR, Schwartz SG, Flynn HW. Endophthalmitis following pars plana vitrectomy for vitreous floaters. Clin Ophthalmol Auckl NZ. 2014;8:1649–53. 10.2147/OPTH.S67855. PubMed PMID: 25210434; PubMed Central PMCID: PMC4155899. PMC4155899 25210434

[8] Zeydanli EO, Parolini B, Ozdek S, Bopp S, Adelman RA, Kuhn F, et al. Management of vitreous floaters: an international survey the European VitreoRetinal Society Floaters study report. Eye Lond Engl. 2020;34(5):825–34. 10.1038/s41433-020-0825-0 . PubMed PMID: 32313173; PubMed Central PMCID: PMC7182575. PMC7182575 32313173

[9] VanderBeek BL, Chen Y, Tomaiuolo M, Deaner JD, Syed ZA, Acharya B, et al. Endophthalmitis Rates and Types of Treatments After Intraocular Procedures. JAMA Ophthalmol. 2024;142(9):827–34. 10.1001/jamaophthalmol.2024.2749. PMC11295066 39088207

[10] Zafar S, Dun C, Cai CX, Schein OD, Makary M, Woreta F. Endophthalmitis after retinal surgery: a 8-year medicare analysis. Retina Phila Pa. 2025;45(10):1908–15. 10.1097/IAE.0000000000004537. PubMed PMID: 40456117. 40456117

[11] Park JC, Ramasamy B, Shaw S, Ling RHL, Prasad S. A prospective and nationwide study investigating endophthalmitis following pars plana vitrectomy: clinical presentation, microbiology, management and outcome. Br J Ophthalmol. 2014;98(8):1080–6. 10.1136/bjophthalmol-2013-304486 . PubMed PMID: 24686917. 24686917

[12] Bhende M, Raman R, Jain M, Shah PK, Sharma T, Gopal L, et al. Incidence, microbiology, and outcomes of endophthalmitis after 111,876 pars plana vitrectomies at a single, tertiary eye care hospital. PLoS ONE. 2018;13(1):e0191173. 10.1371/journal.pone.0191173 . PubMed PMID: 29338030; PubMed Central PMCID: PMC5770060. PMC5770060 29338030

[13] Eifrig CWG, Scott IU, Flynn HW, Smiddy WE, Newton J. Endophthalmitis after pars plana vitrectomy: Incidence, causative organisms, and visual acuity outcomes. Am J Ophthalmol. 2004;138(5):799–802. 10.1016/j.ajo. 2004.06.035 PubMed PMID: 15531315. 15531315

[14] Arya P, Sharma V, Singh P, Thapliyal S, Sharma M. Bacterial endotoxin-lipopolysaccharide role in inflammatory diseases: An overview. Iran J Basic Med Sci. 2025;28(5):553–64. 10.22038/ijbms.2025.82302.17799 . PubMed PMID: 40666174; PubMed Central PMCID: PMC12258786. PMC12258786 40666174

[15] Sridhar J, Kuriyan AE, Flynn HW, Miller D, Endophthalmitis caused by pseudomonas. aeruginosa: clinical features, antibiotic susceptibilities, and treatment outcomes. Retina Phila Pa. 2015;35(6):1101–6. 10.1097/IAE.0000000000000469. PubMed PMID: 25658178. 25658178

[16] Sridhar J, Kuriyan AE, Flynn HW, Smiddy WE, Venincasa VD, Miller D. Endophthalmitis caused by serratia marcescens: clinical features, antibiotic susceptibilities, and treatment outcomes. Retina Phila Pa. 2015;35(6):1095–100. 10.1097/IAE.0000000000000509. PubMed PMID: 25741815. 25741815

[17] Yannuzzi NA, Patel NA, Relhan N, Tran KD, Si N, Albini TA, et al. Clinical features, antibiotic susceptibilities, and treatment outcomes of endophthalmitis caused by *Staphylococcus epidermidis*. Ophthalmol Retina. 2018;2(5):396–400. 10.1016/j.oret.2017.08.025. 31047321

